# Supplementary figures and images for: Combining multisensor images and social network data to assess the area flooded by a hurricane event
Source: PeerJ. 2024 Apr 29;12:e17319. doi: 10.7717/peerj.17319 (PMC11064868; doi:10.7717/peerj.17319)

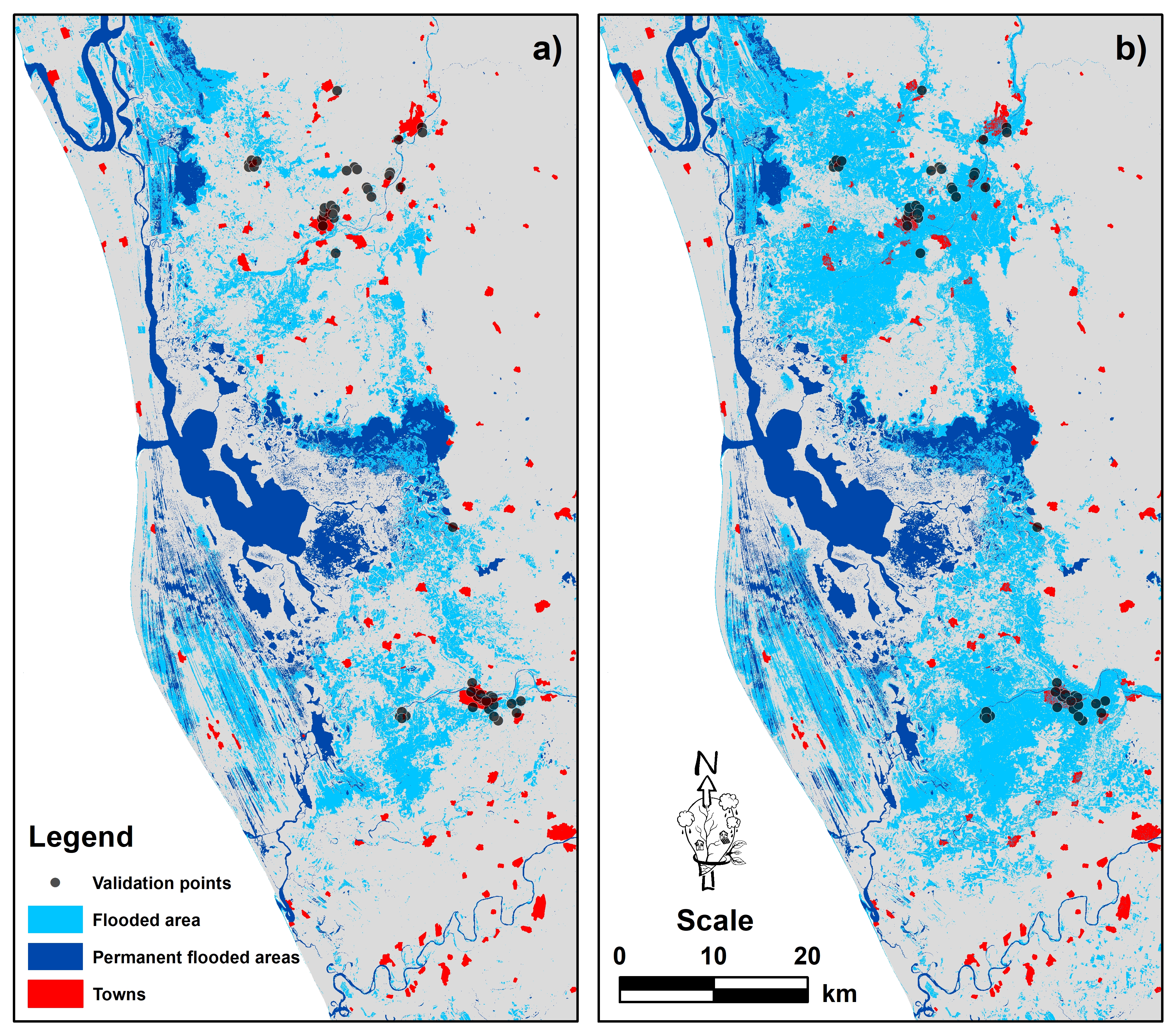

Supplement: Supplemental Information 1 — Map source credit: Images were processed from Copernicus Sentinel-2 data (2018), (C) European Space Agency-ESA. [file peerj-12-17319-s001.jpg]

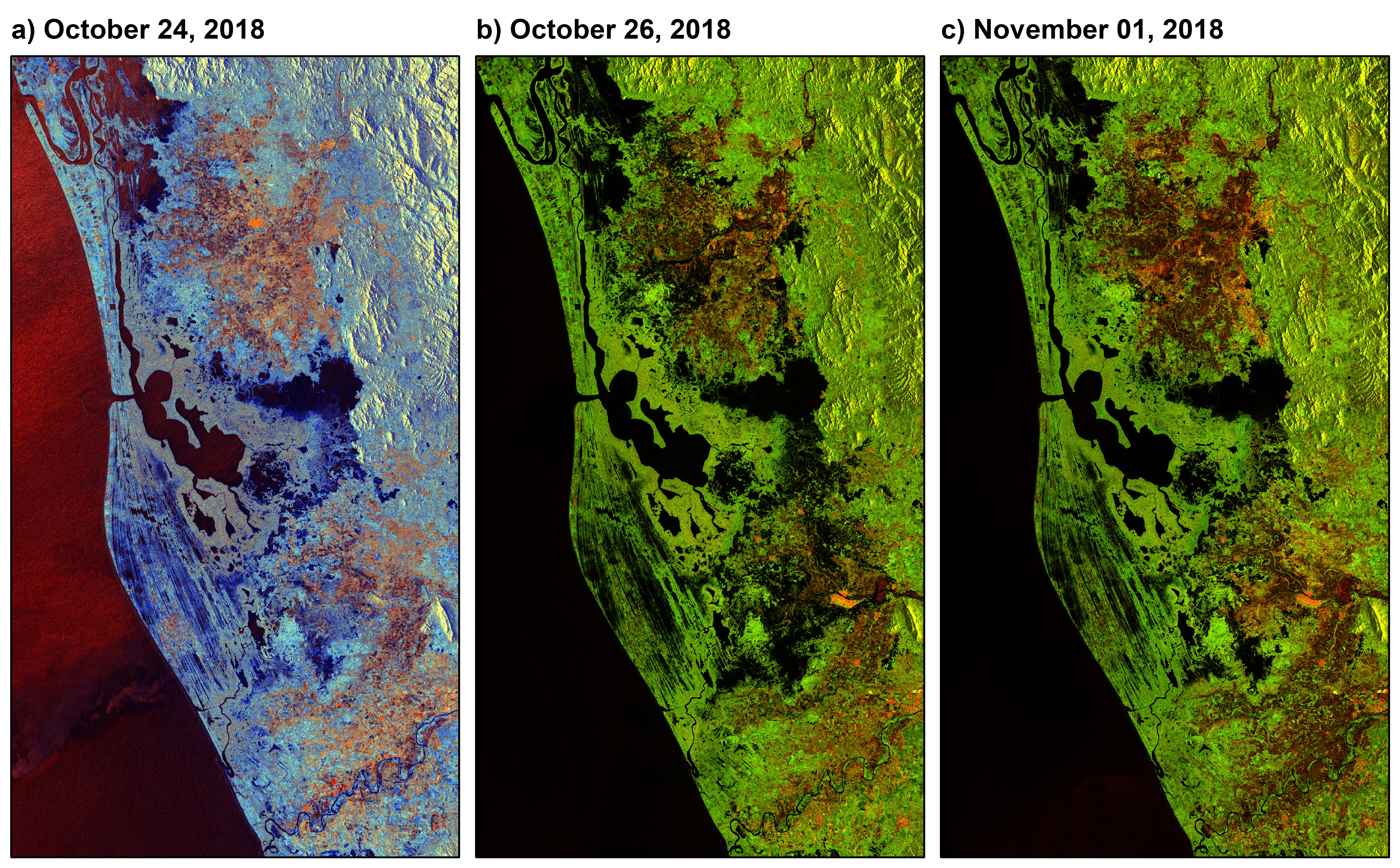

Supplement: Supplemental Information 2 — Map source credit: Images were processed from Copernicus Sentinel-2 data (2018), (C) European Space Agency - ESA. [file peerj-12-17319-s002.jpg]

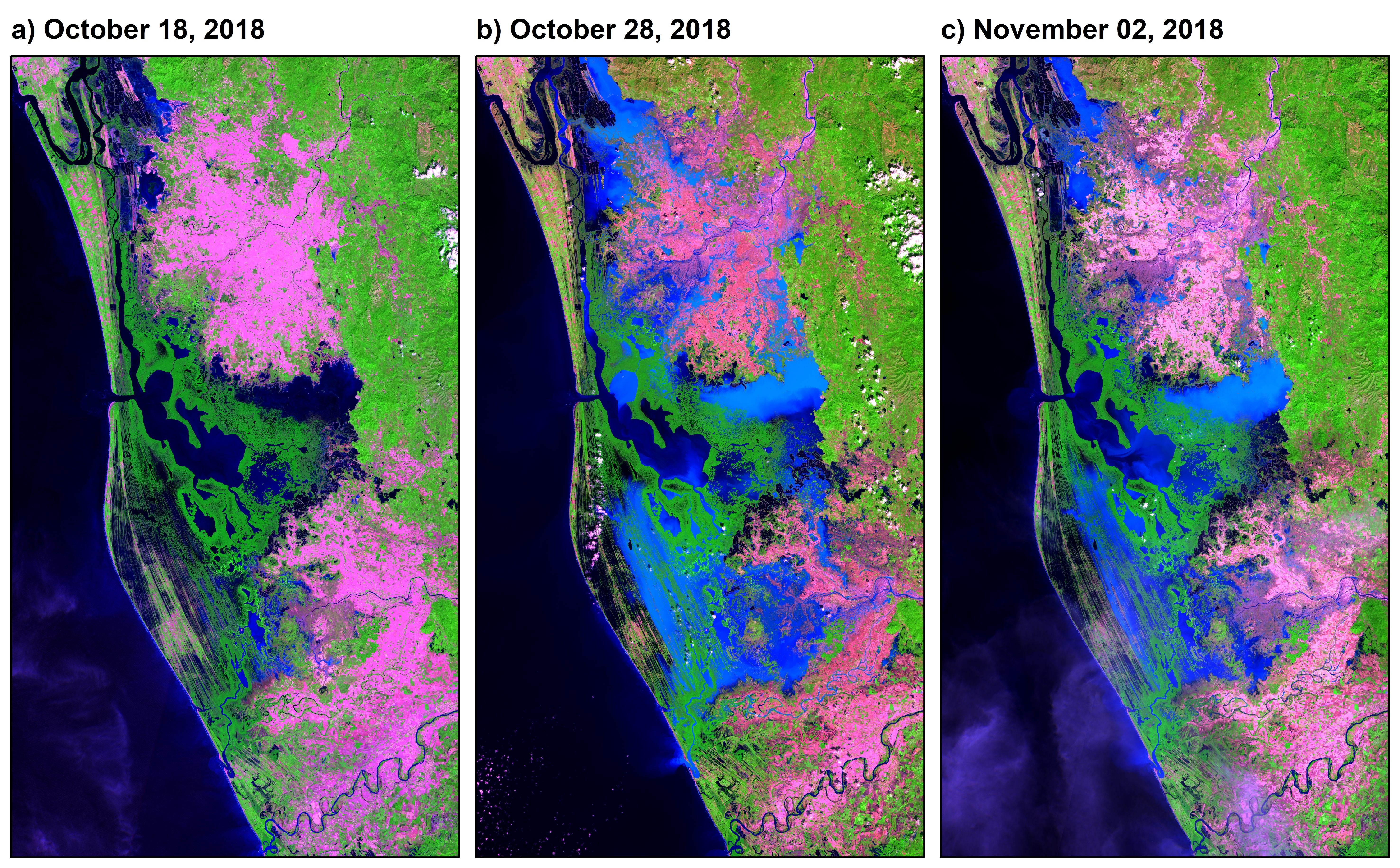

Supplement: Supplemental Information 3 — Map source credit: Images were processed from Copernicus Sentinel-2 data (2018), (C) European Space Agency - ESA. [file peerj-12-17319-s003.jpg]
